# DHZCP Modulates Microglial M1/M2 Polarization *via* the p38 and TLR4/NF-κB Signaling Pathways in LPS-Stimulated Microglial Cells

**DOI:** 10.3389/fphar.2020.01126

**Published:** 2020-07-30

**Authors:** Lu Zhou, Dongsheng Wang, Xinjian Qiu, Weiru Zhang, Zhicheng Gong, Yang Wang, Xia Xu

**Affiliations:** ^1^Department of Integrated Traditional Chinese and Western Medicine, Xiangya Hospital, Central South University, Changsha, China; ^2^Department of General Medicine, Xiangya Hospital, Central South University, Changsha, China; ^3^Department of Pharmacy, Xiangya Hospital, Central South University, Changsha, China

**Keywords:** Dahuang Zhechong Pill (DHZCP), antiinflammatory effects, microglia polarization, p38, TLR4/NF-κB

## Abstract

Intracerebral hemorrhage (ICH) is a disease with a significantly high rate of morbidity, mortality and disability. Inhibition of inflammation is considered a potential strategy for improving the clinical symptoms induced by ICH. The hallmark of neuroinflammation is microglial activation. Microglia can polarize into either the classically activated M1 (proinflammatory) phenotype, exacerbating neuronal damage, or the alternatively activated M2 (antiinflammatory) phenotype, exerting neuroprotection and promoting neuronal recovery. Promoting microglial polarization to the M2 phenotype may be a viable strategy for treating neuroinflammation. Several studies have indicated that promoting blood circulation and removing blood stasis exhibits therapeutic effects on intracerebral hemorrhage. Dahuang Zhechong Pill (DHZCP), a classical recipe that promotes blood circulation and removes blood stasis, has been reported to improve the clinical outcome of ICH. DHZCP has been shown to exert antiinflammatory effects. However, the detailed antiinflammatory mechanism of DHZCP in ICH has rarely been investigated. In this study, DHZCP inhibited lipopolysaccharide (LPS)-induced M1 microglial activation. DHZCP exerted antiinflammatory effects, by inhibiting LPS-induced M1 proinflammatory cytokine (TNF-α and IL-6), and iNOS production and increasing M2 antiinflammatory cytokine (IL-10) production. DHZCP also switched microglial polarization from M1 to M2, as indicated by significantly increased expression of M2 polarization markers (CD209, and CD206) and markedly decreased expression of an M1 polarization marker (CD54). In addition, DHZCP inhibited p38 and TLR4/NF-κB signaling activation, as demonstrated by inhibition of LPS-induced increases in p-p38, TLR4 and nuclear factor kappa B p-65 (NF-κB p-65) protein expression. Taken together, DHZCP modulates microglial M1/M2 polarization *via* the p38 and TLR4/NF-κB signaling pathways to confer antiinflammatory effects.

## Introduction

Intracerebral hemorrhage (ICH) is a disease with a significantly high rate of morbidity, mortality and disability that lacks effective therapies (Wang and Dore, 2007; Zhou et al., 2014; Zeng et al., 2016). Therefore, it is essential and urgent to explore potential therapeutic approaches to improve the clinical outcomes of ICH. A thorough understanding of the pathogenesis of ICH-induced secondary brain injury is crucial, especially inflammatory mechanisms (Wang et al., 2007; Zhou et al., 2014). Evidence suggests that inflammation critically contributes to ICH-induced brain injury, such as hematoma and neuron injury (Zhang et al., 2006; Zhou et al., 2014), and in return, hematoma triggers inflammatory signaling through microglial activation (Zhou et al., 2014). Inhibition of inflammation is a potential strategy for treating ICH (Fang et al., 2013). To investigate the mechanisms of ICH-induced inflammatory injury, microglial activation is a point of concern for researchers (Zhou et al., 2014).

In China, traditional Chinese medicine (TCM) formulas combined with Western medicine are widely used to treat ICH. Different TCM prescriptions are used during the pathological process of ICH, such as Buyang Huanwu Decoction (Li P. et al., 2019), naoyian granule (Xiao et al., 2004), and Xingnaojing injection (Peng et al., 2014), which contain herbs that promote blood circulation and remove stasis. Promoting blood circulation and removing stasis is an essential treatment for ICH in TCM as blood stasis syndrome is part of the pathological process of ICH (Li et al., 2015). Several studies on the therapeutic effects of promoting blood circulation and removing blood stasis on ICH have been undertaken. Clinical research by Sun Guozhu showed that promoting blood circulation and removing blood stasis exhibited therapeutic effects on hemorrhagic apoplexy, significantly changing the conscious state and motor function of patients (Sun, 2003). A meta-analysis assessing nine randomized-controlled clinical trials with 798 individuals indicated that promoting blood circulation and removing blood stasis for acute ICH improved neurological function, reduced the volume of brain hematoma and cerebral edema, and decreased the mortality and disability rate (Li et al., 2015). Moreover, Zeng et al. conducted a randomized, 13 hospital, placebo-controlled clinical trial to further confirm whether removing blood stasis is a safe and effective treatment for hypertensive ICH (Zeng et al., 2016).

Dahuang Zhechong Pill (DHZCP) is a famous classical Chinese prescription originating from Jin-Gui-Yao-Lue (Synopsis of Prescriptions of the Golden Chamber) in the Han dynasty, and contains 12 herbs: Eupolyphaga Seu Steleophaga, *Rheum palmatum* L. (rhubarb), *Scutellaria baicalensis* Georgi. (Scutellariae), *Glycyrrhiza glabra* L. (licorice), *Prunus persica* (L.) Batsch. (peach kernel), *Armeniaca dasycarpa* (Ehrh.) Borkh. (almond), *Paeonia officinalis* L.(Lactiflora), *Rehmannia glutinosa* (Gaertn.) DC. (Rehmannia), *Toxicodendron vernicifluum* (Stokes) F.A. Barkley. (dry paint), *Tabanus bivittatus* Matsumura, Hirudo, and *Holotrichia diomphalia* Bates (Zhang et al., 2009; Wu et al., 2018). DHZCP possesses pharmacodynamic actions, such as promoting blood circulation and removing stasis, and has been widely applied in the treatment of blood stasis, hepatic diseases and atherosclerosis (Xing et al., 2012). DHZCP, a classical recipe that promotes blood circulation and removes blood stasis, improves the treatment efficacy of ICH (Wang et al., 2007). Previous research demonstrated that DHZCP downregulated secretion of the proinflammatory cytokines, tumor necrosis factor-α (TNF-α) and interleukin-13 (IL-13), by decreasing the phosphorylation of p38 in CCl_4_-intoxicated rats (Cai et al., 2010). This indicates that inhibiting the inflammatory response may reveal the therapeutic mechanism of DHZCP in ICH. However, the detailed mechanisms by which DHZCP mediated antineuroinflammation are not clearly illustrated. The hallmark of neuroinflammation is microglial activation (Zhang et al., 2019). In response to neuroinflammation, microglia become activated and undergo changes into two distinct functional polarization states: the classically activated M1 phenotype (proinflammatory) exacerbates neuronal damage, and the alternatively activated M2 phenotype (antiinflammatory) exerts neuroprotection (Zhang et al., 2019). M1 cells express surface marker such as CD54, and produce pro-inflammatory cytokines such as TNF-α and IL-6, while M2 cells express C-type lectins CD206 and CD209, and produce antiinflammatory cytokines, such as IL-10 (Cho and Choi, 2017). Promoting microglial polarization to the M2 phenotype could be a viable strategy for the treatment of neuroinflammation (Zhang et al., 2019).

Thus, we hypothesized that DHZCP mediated antineuroinflammation modulates microglial M1/M2 polarization. To test this hypothesis, lipopolysaccharide (LPS)-activated microglial cells were examined to determine whether and how DHZCP participates in microglial activation. In addition, we investigated whether DHZCP promoted microglial M1/M2 polarization *via* the p38 and TLR4/NF-κB signaling pathways. These findings provide a theoretical basis for the clinical application of DHZCP.

## Materials and Methods

### Materials

DHZCP was purchased from Beijing Tong Ren Tang Co., Ltd. (batch no: Z11020002, 3 g/pill). The BV-2 cell (a mouse microglial cell line) was purchased from Basal Institute Cell Center, of Wuhan Union Hospital (PR China). Dulbecco’s modified Eagle’s medium (DMEM), fetal bovine serum (FBS), and 0.25%trypsin-EDTA were obtained from Gibco Life Technologies™. TRIzoL reagent and SYBRgreen PCR mix were purchased from Invitrogen. The RevertAid First Strand cDNA Synthesis Kit was purchased from Beijing ComWin Biotech Co., Ltd. The primers were purchased from Sangon Biotech (Shanghai) Co.,Ltd. Taq polymerase, dNTPs and DL2000 DNA MAPKer were purchased from Genstar. Protease inhibitor cocktail tablets was supplied by Merck (Merck, Germany) and phosphatase inhibitor cocktail tablets were supplied by Roche (Roche, Indianapolis, IN, USA). Lipopolysaccharide (LPS), and interleukin-4 were obtained from Beijing Chemical Reagent Company. Anti-p-65 antibody (#6956) was purchased from Cell Signaling Technology (USA). Anti-PCNA antibody (10205-2-AP), anti-TLR4 antibody (19811-1AP), anti-p38 antibody (14064-1-AP), Alexa Fluor 594-conjugated goat anti-mouse IgG (SA00006-3), HRP goat anti-mouse IgG and HRP goat anti-rabbit IgG were obtained from Proteintech Group, Inc. (USA). Anti-phospho-p38 antibody (Ab195049), anti-CD206 antibody (Ab8918), anti-CD54 antibody (Ab119871) and Goat anti-rat IgG H&L (Alexa Fluor^®^ 488) (ab150157) were obtained from Abcam. Anti-GAPDH antibody (Ap0063) was obtained from Bioworld Technology, Inc. The PierceTM BCA protein assay kit was purchased from Thermo Fisher Scientific.

### Preparation of Serum Containing DHZCP

Male Sprague Dawley rats, weighing 200 ± 20 g, were provided by the Animal Experimental Center in Kaifu District (Changsha, China), and caged (3 rats per standard cage) with free access to food and tap water under strictly controlled conditions (room temperature of 25°C ± 2°C, relative humidity of 50% ± 10% and a 12-h light/dark cycle). This research was strictly conducted in accordance with the Regulations for the Administration of Affairs Concerning Experimental Animals (1988), which were approved by the Animal Experimental Center for Central South University (Changsha, China). The rats were allowed to adapt to the new environment for 3 days before the commencement of this experiment.

The rats were randomly divided into three groups and then intragastrically administrated saline or DHZCP for 7 days (twice per day): the control group was treated with saline, the low-dose DHZCP group received 0.25 g kg^-1^d^-1^ DHZCP, and the high-dose DHZCP group received 0.75 g kg^-1^ d^-1^ DHZCP. The rats were sacrificed 1 h later after the last administration. Blood samples were collected aseptically from the abdominal aorta of rats and then kept at room temperature for approximately 1 h. Following centrifugation at 3,000 rpm min^-1^ for 12 min at 4°C, serum samples were acquired. Following two filtrations with a 0.22 µm cellulose acetate membrane, the sera were decanted, incubated in 56°C water for 30 min and then stored at −20°C until use.

### Cell Culture

Microglia were cultured in a humidified incubator with a 5% CO_2_ atmosphere and 95% air at 37°C. The medium used for cell culture, which was changed regularly, consisted of 89% DMEM, 0.5% penicillin (100 units ml^-1^), 0.5% streptomycin (100 µg ml^-1^) and 10% FBS.

### Experimental Procedures

Microglial cells were seeded into 6-well plates. After 24 h of incubation at 80% relative humidity, the cell culture media were discarded and replaced with the new culture system. Then, the cells were divided into subgroups. DMEM containing 10% normal serum was added to the cultures in the control group, DMEM containing both 10% normal serum and stimulants (100 ng/ml LPS or 20 ng/ml IL-4) was added to the cultures in the model group, and DMEM containing 10% of rat serum that had been treated with different doses of DHZCP and the abovementioned stimulants was added to the cultures in the DHZCP groups. Cells in the DHZCP groups were preincubated with serum containing LPS for 24 h, followed by incubation with serum containing DHZCP for an additional 24 h. Cells in the control and model control groups were incubated with the corresponding serum for 24 h and harvested on the similar day as the DHZCP groups.

### Real-Time PCR Analysis

Total RNA was extracted with TRIzol reagent in accordance with the manufacturer’s instructions. The concentration of RNA was quantified, and the quality of the RNA was assessed through the ratio of the absorbances at 260 and 280 nm. Subsequently, RNA was reverse transcribed to cDNA using a RevertAid First Strand cDNA Synthesis kit, and then the cDNA was amplified using a SYBRgreen PCR master mix kit. The sequences of the primers are shown in **Table 1**. The reactions were conducted with a total reaction volume of 30 µl containing 1 µl template. The RT-PCR program was set as follows: 95°C for 10 min, 40 cycles of 95°C for 15 s, and 60°C for 50 s. The relative mRNA expression was analyzed by the 2^-ΔΔCt^ method.

**Table 1:** The RT-PCR oligonucleotide primers.

Gene	Primer	Sequence(5’to3’)	PCR product(bp)
iNOS	Forward Reverse	AGGGAATCTTGGAGCGAGTT GCAGCCTCTTGTCTTTGACC	105	
IL-10	Forward Reverse	CTTGCACTACCAAAGCCACA TGATCCTCATGCCAGTCAGT	105	
IL-6	Forward Reverse	CCGGAGAGGAGACTTCACAG CATTTCCACGATTTCCCAGA	106	
GAPDH	Forward Reverse	CGGCAAATTCAACGGCACA GGTCTCGCTCCTGGAAGATGG	86	

### Enzyme-Linked Immunosorbent Assay

The cell culture medium was collected and the levels of TNF-α, IL-10 and IL-6 were measured by commercial Enzyme-Linked Immunosorbent Assay (ELISA) Kit (Invitrogen) according to the manufacturerˊs instructions. Briefly, medium was added and incubated in biotin and streptavidin-HRP buffer at 37°C for 1 h. Following the substrate reaction, color development was stopped by the stop buffer, and the OD was read at 450 nm with a microplate reader (MB-530, Heales, China) within 20 min.

### Flow Cytometry

The cells were digested from the culture dish, washed with phosphate-buffered saline (PBS) twice and then adjusted to a concentration of 10^6^ cells per ml in 100μl PBS. Then, CD209-APC was added. Following incubation in the dark at room temperature for 15min, and washing three times with PBS, the expression of CD209 was detected by a FACSCalibur flow cytometer (BD Biosciences, Burlington, USA).

### Immunofluorescence

Cells were seeded on glass coverslips and incubated with the corresponding serum. Then, the coverslips were fixed with 4% paraformaldehyde for 30 min at 37°C, rinsed three times with PBS, treated with 0.1% Triton X-100 at 37°C for 30 min and subsequently washed three times with PBS. Following blocking with 5% bovine serum albumin for 1 h at 37°C, the coverslips were incubated with diluted primary antibody [CD206 (1:100), CD54 (1:100)] overnight at 4°C. After washing three times with PBS, the coverslips were incubated with Alexa Fluor 594-conjugated goat anti-mouse IgG and goat anti-rat IgG H&L for 1 h at 37°C in the dark. The nuclei were stained with DAPI, and the cells were imaged under a fluorescence microscope (Motic, BA410E).

### Western Blot Analysis

Cells were lysed in RIPA buffer containing 50 mM Tris-HCl with PH 8.0, 150 mM NaCl, 1% Triton X-100, 0.25% sodium deoxycholate, and 1 mM EDTA supplemented with a protease inhibitor cocktail and a phosphatase inhibitor cocktail on ice, and centrifuged to separate the proteins. The protein concentrations were determined using a BCA protein assay kit. Subsequently, the proteins were separated by SDS-PAGE, transferred onto PVDF membranes and blocked with 5% nonfat milk in Tris-buffered saline with 0.1% Tween-20 (TBST). The membranes were incubated overnight at 4°C with primary antibodies against nuclear factor kappa B p-65 (1:1000 dilution), PCNA (1:2,000 dilution), TLR (1:1,000 dilution)4, p-38 (1:1,000 dilution), p-p38 (1:1,000 dilution) and GAPDH (1:5,000 dilution). Following incubation for 1 h at room temperature with anti-mouse horseradish peroxidase-conjugated secondary antibodies (1:4,000 dilution) for p-65 and with anti-rabbit horseradish peroxidase-conjugated secondary antibody (1:6,000 dilution) for PCNA, TLR4, p-38, p-p38 and GAPDH, the proteins were detected with an enhanced chemiluminescence (ECL) kit and imaged with a ChemiDoc™ XRS imaging system (Bio-Rad Laboratories, USA). The protein bands were scanned by Image Lab software (version 4.0, Bio-Rad, USA), and quantitative analysis was performed with Quantity One Software. The optical density was normalized to PCNA or GAPDH.

### Statistical Analysis

The data are presented as the means ± S.D. All data were analyzed with GraphPad Prism 6.0 software. Mean values between groups were compared by one-way analysis of variance (ANOVA), and a value of P<0.05 was considered to be statistically significant.

## Results

### Effects of Rat Serum Containing DHZCP on LPS-Induced Production of Inflammatory Factors in Microglia

**Figure 1:**
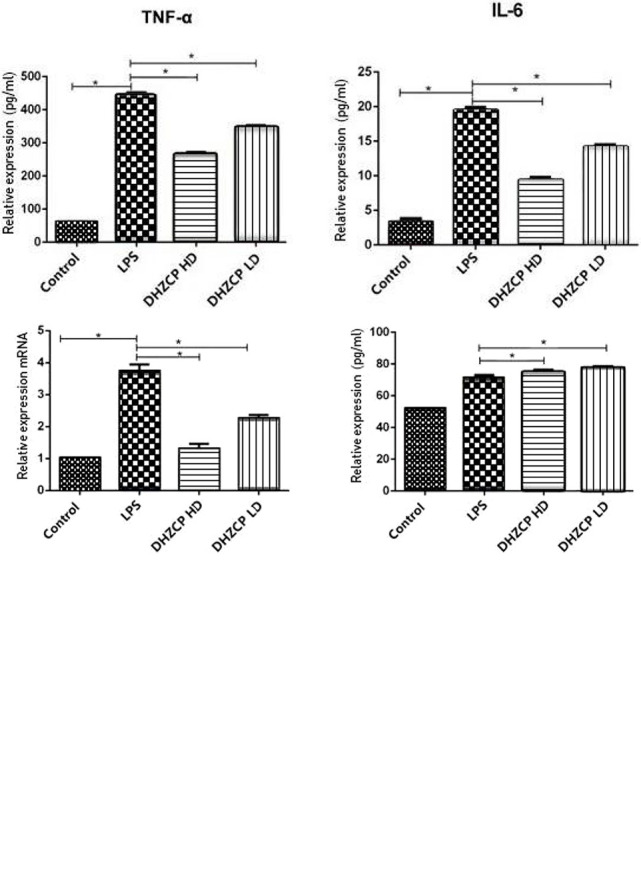
mRNA expression of IL-10, IL-6 and iNOS in microglia was detected by PCR, and the production of IL-10, IL-6 and TNF-α was measured by ELISA. The data show mean ± S.D. statistically differences between groups were shown as follows: *P < 0.05, n=3. Control, the control group in which cells were cultured in Dulbecco’s modified Eagle’s medium (DMEM) containing 10% normal serum for 24 h; lipopolysaccharide (LPS), the LPS model control group in which cells were cultured in DMEM containing both 10% normal serum and stimulant (100 ng/ml LPS) for 24 h; DHZCP HD, DHZCP high dose group in which cells were preincubated with serum containing LPS for 24 h, followed incubation with DMEM containing 10% serum from rats that were treated with a high dose of DHZCP for an additional 24 h; DHZCP LD, DHZCP low dose group in which cells were incubated with serum containing LPS for 24 h, followed DMEM containing 10% serum from rats that were treated with a low dosage DHZCP for an additional 24 h.

To assess the effects of rat serum containing DHZCP on LPS-induced mRNA expression and production of inflammatory cytokines in microglia, mRNA expression of IL-10, IL-6, and inducible nitric oxide synthase (iNOS) was detected by PCR, and the production of IL-10, IL-6, and TNF-α was measured by ELISA. As shown in **Figure 1**, the production of M1 proinflammatory cytokines (TNF-α and IL-6) and iNOS was increased significantly in the LPS group compared to that in the control group, and the production of the M2 antiinflammatory cytokine (IL-10) was not statistically different, as expected. LPS-induced M1 proinflammatory cytokine (TNF-α and IL-6) and iNOS production was inhibited in the DHZCP groups, while M2 antiinflammatory cytokine (IL-10) production was significantly increased. These results suggest that LPS increased the production of proinflammatory factors and that serum containing DHZCP exerted antiinflammatory effects on LPS-activated microglial cells.

### Effects of Rat Serum Containing DHZCP on the Expression of CD209 in LPS-Treated Microglial Cells

**Figure 2:**
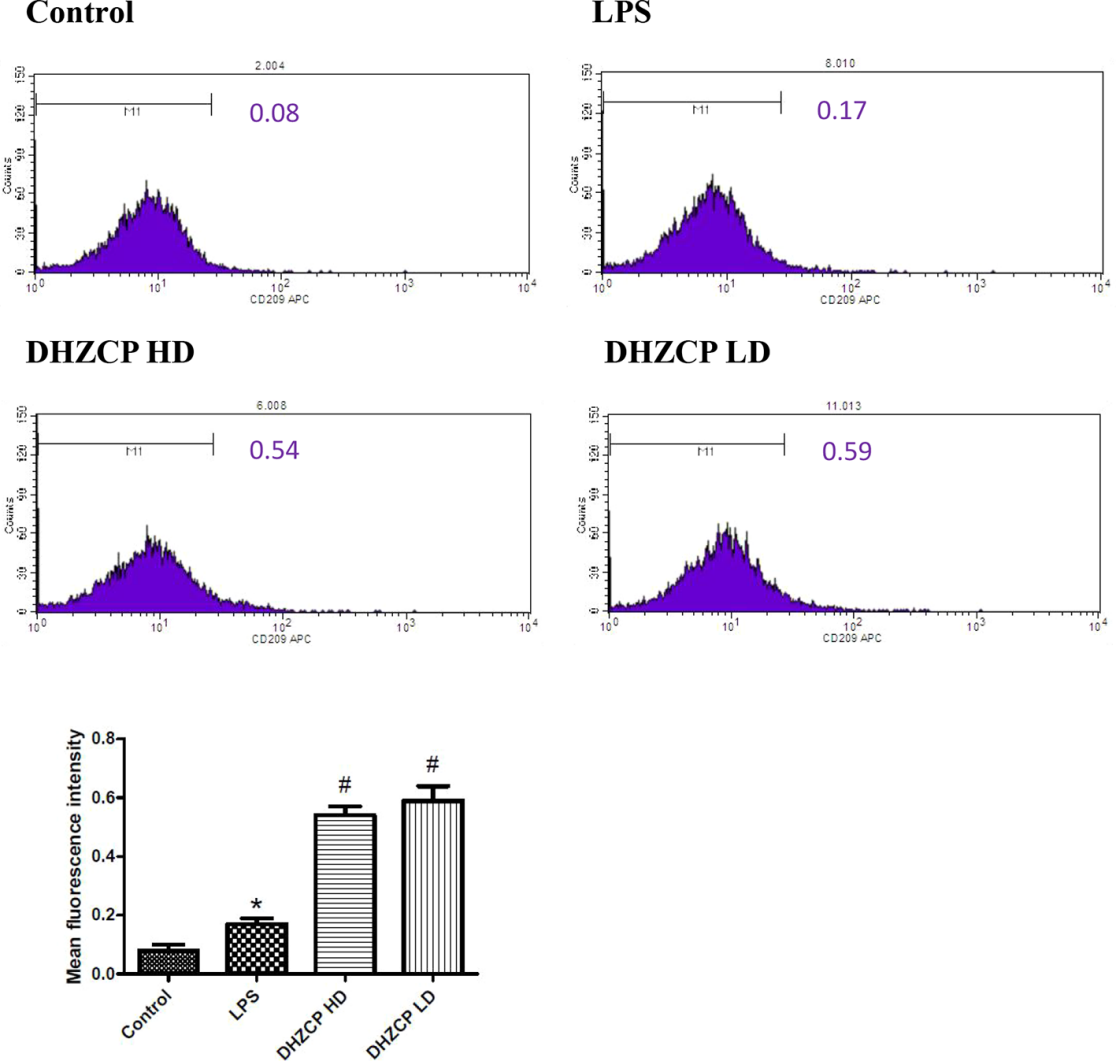
The expression level of CD209 in microglia was measured by flow cytometry. The data (mean fluorescence intensity: geometric mean) show mean ± S.D. statistically differences between groups were shown as follows: *P < 0.05, vs. Control group; ^#^P < 0.05, vs. lipopolysaccharide (LPS) group, n=3. Control, the control group in which cells were cultured in Dulbecco’s modified Eagle’s medium (DMEM) containing 10% normal serum for 24 h; LPS, the LPS model control group in which cells were cultured in DMEM containing both 10% normal serum and stimulant (100 ng/ml LPS) for 24 h; DHZCP HD, DHZCP high dose group in which cells were preincubated with serum containing LPS for 24 h, followed incubation with DMEM containing 10% serum from rats that were treated with a high dose of DHZCP for an additional 24 h; DHZCP LD, DHZCP low dose group in which cells were incubated with serum containing LPS for 24 h, followed DMEM containing 10% serum from rats that were treated with a low dosage DHZCP for an additional 24 h.

To explore whether rat serum containing DHZCP promoted M2 polarization of LPS-treated microglial cells, the expression level of CD209 was measured by flow cytometry. As presented in **Figure 2**, though the differences between groups were relatively small, the expression of CD209 was slightly increased in the DHZCP groups compared to the LPS and control groups. This suggests that serum containing DHZCP could promote M2 polarization of microglia.

### Rat Serum Containing DHZCP Switched LPS-Induced Microglial Polarization From M1 to M2

**Figure 3:**
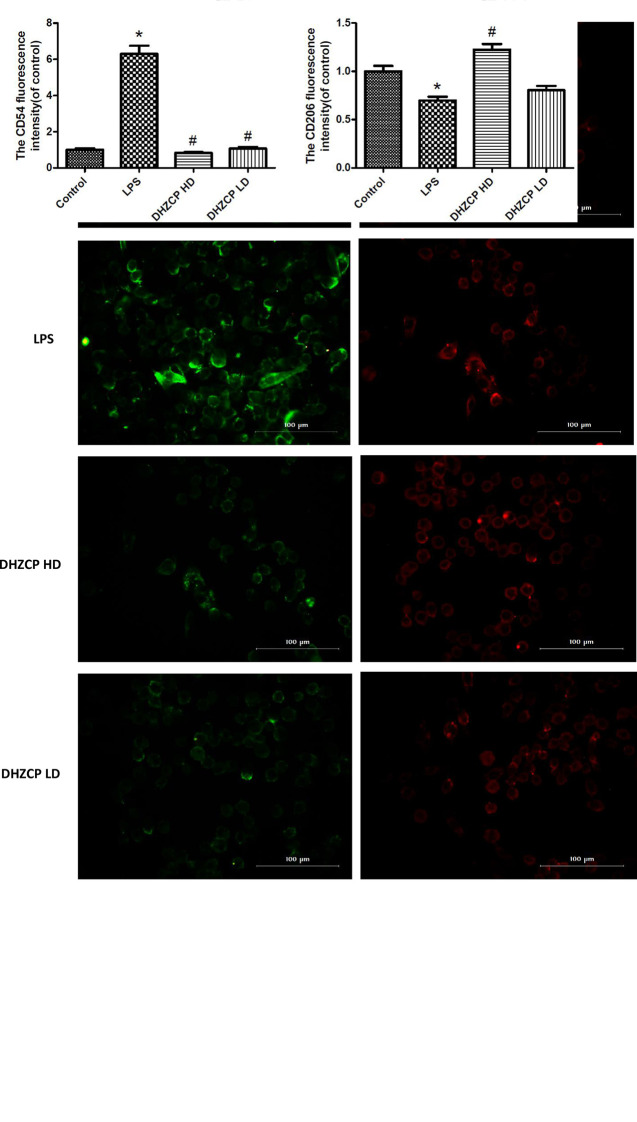
The expression of CD54 (green fluorescence) and CD206 (red fluorescence) in microglia were detected by immunofluorescence (scale bar: 100μm) and the fluorescence intensity was analyzed by Image J 1.8.0 version. The data show mean ± S.D. statistically differences between groups were shown as follows: *P < 0.05, vs. Control group; ^#^P < 0.05, vs. lipopolysaccharide (LPS) group, n=3. Control, the control group in which cells were cultured in Dulbecco’s modified Eagle’s medium (DMEM) containing 10% normal serum for 24 h; LPS, the LPS model control group in which cells were cultured in DMEM containing both 10% normal serum and stimulant (100 ng/ml LPS) for 24 h; DHZCP HD, DHZCP high dose group in which cells were preincubated with serum containing LPS for 24 h, followed incubation with DMEM containing 10% serum from rats that were treated with a high dose of DHZCP for an additional 24 h; DHZCP LD, DHZCP low dose group in which cells were incubated with serum containing LPS for 24 h, followed DMEM containing 10% serum from rats that were treated with a low dosage DHZCP for an additional 24 h.

To further evaluate the effect of serum containing DHZCP on microglial polarization, the expression of CD54 and CD206 was detected by immunofluorescence. CD54 is an M1 polarization marker, while CD206 represents M2 microglial polarization. Similar to the flow cytometry results, immunofluorescence staining (**Figure 3**) revealed that the expression of CD54 (green fluorescence) in the DHZCP groups was markedly decreased compared to that in the LPS group; however, CD206 expression (red fluorescence) in the DHZCP groups was significantly increased compared to that in the LPS and the control groups. These results indicate that serum containing DHZCP switched the inflammatory M1 phenotype to the antiinflammatory M2 phenotype.

### Effects of Rat Serum Containing DHZCP on the p38 and TLR4/NF-κB Signaling Pathways in LPS-Induced Microglial Cells

**Figure 4:**
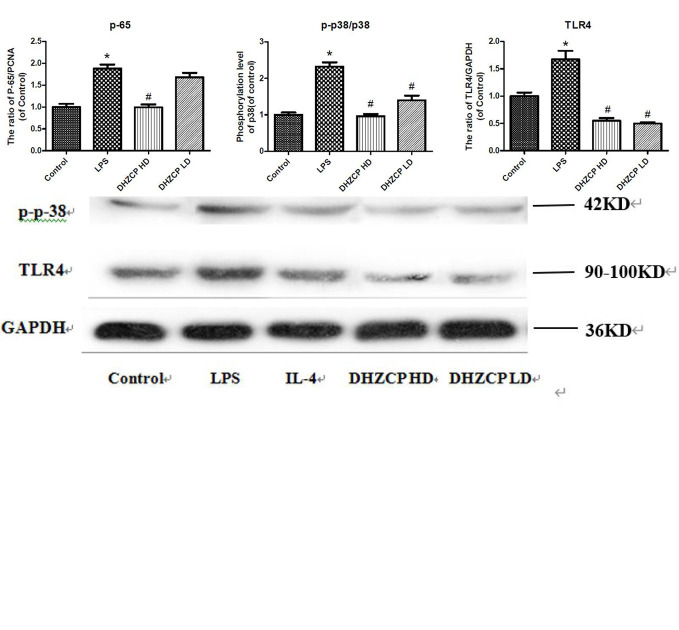
The expression levels of P-65, P-38, P-P-38 and TLR4 in microglia were visualized by Western blotting and quantified using Image J. The data in the figures was presented as the means ± S.D. (n=3) for three independent experiments. Statistically significant differences between groups were shown as follows: *P < 0.05, vs. Control group; ^#^P < 0.05, vs. lipopolysaccharide (LPS) group. Control, the control group in which cells were cultured in Dulbecco’s modified Eagle’s medium (DMEM) containing 10% normal serum for 24 h; LPS, the LPS model control group in which cells were cultured in DMEM containing both 10% normal serum and stimulant (100 ng/ml LPS) for 24 h; Dahuang Zhechong Pill (DHZCP) HD, DHZCP high dose group in which cells were preincubated with serum containing LPS for 24 h, followed incubation with DMEM containing 10% serum from rats that were treated with a high dose of DHZCP for an additional 24 h; DHZCP LD, DHZCP low dose group in which cells were incubated with serum containing LPS for 24 h, followed DMEM containing 10% serum from rats that were treated with a low dosage DHZCP for an additional 24 h.

Microglia were preincubated with LPS for 24 h and then incubated for an additional 24 h with or without serums from rats that had been treated with different concentrations of DHZCP. To observe the effects of rat serum containing DHZCP on p38 and TLR4/NF-κB signaling pathways in the presence of LPS stimulation, the protein expression levels of p38, p-p38, TLR4, and p-65 in microglial cells were determined by Western blotting. The results showed (**Figure 4**) that LPS stimulation visibly upregulated p-p38, TLR4, and p-65 protein expression compared to that of the control group, which was obviously attenuated after treatment with the serum containing DHZCP. This indicated that serum containing DHZCP inhibited LPS-induced increases in p-p38, TLR4, and p-65 protein expression.

## Discussion

Microglia, the resident macrophages of the brain, constantly survey the microenvironment and produce factors that influence surrounding astrocytes and neurons (Huang et al., 2019). Microglia can polarize into either the classically activated M1 phenotype or the alternatively activated M2 phenotype (Zhang et al., 2019). Generally, increased production of proinflammatory factors that result in neuronal damage, such as TNF-α and IL-6, and upregulation of iNOS and CD54 are considered markers of the activated M1 phenotype (Qin et al., 2016; Zhang et al., 2019). Conversely, upregulation of antiinflammatory mediators that exert protective effects, including CD206, CD209 and IL-10, is characterized as the activated M2 phenotype (Durafourt et al., 2012; Qin et al., 2016; Chen et al., 2017; Zhang et al., 2019). LPS activates microglia toward the M1 phenotype and increases the secretion of proinflammatory factors (Qiu et al., 2020), which is consistent with our results in the present study. Previous studies have showed that shifting the microglial phenotype from M1 to M2 while inhibiting M1 activation is more beneficial for suppressing neuroinflammation than simply inhibiting M1 activation (Zhang et al., 2019). Interestingly, this study demonstrated that DHZCP attenuated the increased expression of M1 phenotype markers (TNF-α, iNOS, IL-6 and CD54) and upregulated the expression of M2 phenotype markers (IL-10 and CD209) in LPS-stimulated microglia, indicating that DHZCP inhibits M1 activation while promoting microglial polarization from M1 to M2, thus inhibiting microglia-mediated neuroinflammation.

Evidence of the signaling events underlying DHZCP-mediated microglial polarization indicates the involvement of the p38 and TLR4/NF-κB signaling pathways. The p38 pathway, a mitogen-activated protein kinase (MAPK) signaling pathway, regulates the production of proinflammatory factors in activated microglia and critically contributes to allow ecto-5’-nucleotidase to modulate microglial M1/M2 polarization (Zhang et al., 2019). TLR4, a Toll-like receptor (TLR), is critical for ICH-induced inflammatory injury, leading to microglial activation in cultured microglia and an accompanying increase in the release of proinflammatory cytokines (Zhou et al., 2014). Although MAPK and TLRs consist of many subtypes, due to limited conditions, the p38 and TLR4/NF-κB signaling pathways were chosen for investigation in this study for the following reasons: First, the two signaling pathways have been widely reported to regulate the inflammatory response and are associated with microglial activation and subsequent release of proinflammatory cytokines (Zhou et al., 2014; Zhang et al., 2019); Second, DHZCP decreased the production of TNF-α and IL-13 *via* downregulating p38 phosphorylation in CCl_4_-intoxicated rats (Cai et al., 2010); and previous research demonstrated that TLR4 mediates ICH-induced inflammation through the MyD88/TRIF signaling pathways and ultimately regulates the production of inflammatory cytokines *via* NF-κB activation, while TLR2 triggers only the MyD88 signaling pathway and its expression and function in ICH remain to be investigated (Zhou et al., 2014). In addition, in response to LPS, TLR4 activates NF-κB and MAPKs through TIRAP-MyD88 adaptors, leading to the production of cytokines and other proinflammatory proteins (Bruscia et al., 2011). In this study, as DHZCP attenuated the LPS-induced increase in the phosphorylation of p38, the expression of TLR4 and NF-κB p-65, and the production of proinflammatory factors (TNF-α, iNOS and IL-6), we hypothesize that DHZCP inhibits the p-38 and TLR/NF-κB signaling pathways to exert its antiinflammatory effects.

Evidence has shown that promoting blood circulation and removing stasis play important roles in the treatment of inflammation (Ma et al., 2007). DHZCP contains herbs (hirudo, tabanus, peach kernel, and rhubarb) that play critical roles in promoting blood circulation and removing blood stasis (Wei et al., 2015), and the antiinflammatory effects of its active components have been demonstrated. Hirudin, a polypeptide originally obtained from hirudo, reduces leukocyte accumulation and shifts microglia toward the M2 phenotype, which contributes to a reduction in neuroinflammation and an improvement in the outcome of ICH (Li X. et al., 2019). Amygdalin, a compound that is found in peach kernel, exerts antiinflammatory effects by inhibiting LPS-induced mRNA expression of iNOS in mouse BV2 cells (Yang et al., 2007). Rhubarb possesses many actions, including neuroprotective and antiinflammatory properties, and has been used to treat ICH (Wang et al., 2016; Liu et al., 2019). Rhubarb-originated rhein can cross the blood-brain barrier after rhubarb administration, accumulate in the brain and exert neuroprotective effects (Xu et al., 2017). In addition, rhein hydrogel alleviates neuroinflammation by inhibiting the nuclear translocation of p65 in TLR4/NF-κB signaling pathway in LPS-stimulated BV2 microglia (Zheng et al., 2019). These active constituents are associated with the antiinflammatory effects of DHZCP.

In conclusion, our results suggest that DHZCP modulates microglial M1/M2 polarization *via* the p38 and TLR4/NF-κB signaling pathways to confer antiinflammatory effects. These findings provide a basis for the use of DHZCP in neuroinflammation-related conditions. However, further studies, including animal experiments, should be performed, and more clinical evidence should be collected to verify the protective effects of DHZCP against neuroinflammation. Adverse reactions in clinical treatment should be intensively monitored for safety considerations.

## Conclusion

DHZCP mediates antineuroinflammatory effects in LPS-stimulated microglial cells. The protective effects of DHZCP against neuroinflammation are conferred by promoting microglial M1/M2 polarization *via* inhibition of p38 and TLR4/NF-κB signal activation.

## Data Availability Statement

All datasets presented in this study are included in the article/supplementary material.

## Ethics Statement

The animal study was reviewed and approved by the Regulations for the Administration of Affairs Concerning Experimental Animals (1988), which were approved by the Animal Experimental Center for Central South University (Changsha, China).

## Author Contributions

XX took part in the design of the study. XX conducted the experiments. LZ wrote the main manuscript text. All authors participated in performing the laboratory analyses and interpreting the data. All authors contributed to the article and approved the submitted version.

## Funding

This study was supported by National Natural Science Foundation of China (No.81770739), the Clinical Big Data System Construction Project of Central South University (No.46) and Hunan Key Laboratory of Liver viscercal manifestation (No.420010087).

## Conflict of Interest

The authors declare that the research was conducted in the absence of any commercial or financial relationships that could be construed as a potential conflict of interest.
